# Heritability of pulmonary function estimated from pedigree and whole-genome markers

**DOI:** 10.3389/fgene.2013.00174

**Published:** 2013-09-09

**Authors:** Yann C. Klimentidis, Ana I. Vazquez, Gustavo de los Campos, David B. Allison, Mark T. Dransfield, Victor J. Thannickal

**Affiliations:** ^1^Division of Epidemiology and Biostatistics, Mel and Enid Zuckerman College of Public Health, University of ArizonaTucson, AZ, USA; ^2^Department of Biostatistics, Section on Statistical Genetics, University of Alabama at BirminghamBirmingham, AL, USA; ^3^Office of Energetics, School of Public Health, University of Alabama at BirminghamBirmingham, AL, USA; ^4^Allergy and Critical Care Medicine, Department of Medicine, Division of Pulmonary, University of Alabama at BirminghamBirmingham, AL, USA

**Keywords:** FEV1, FVC, FEV1/FVC, heritability, pulmonary function, genetic

## Abstract

Asthma and chronic obstructive pulmonary disease (COPD) are major worldwide health problems. Pulmonary function testing is a useful diagnostic tool for these diseases, and is known to be influenced by genetic and environmental factors. Previous studies have demonstrated that a substantial proportion of the variation in pulmonary function phenotypes can be explained by familial relationships. The availability of whole-genome single nucleotide polymorphism (SNP) data enables us to further evaluate the extent to which genetic factors account for variation in pulmonary function and to compare pedigree- to SNP-based estimates of heritability. Here, we employ methods developed in the animal breeding field to estimate the heritability of forced expiratory volume in one second (FEV_1_), forced vital capacity (FVC), and the ratio of these two measures (FEV_1_/FVC) among subjects in the Framingham Heart Study dataset. We compare heritability estimates based on pedigree-based relationships to those based on genome-wide SNPs. We find that, in a family-based study, estimates of heritability using SNP data are nearly identical to estimates based on pedigree information, and range from 0.50 for FEV_1_ to 0.66 for FEV_1_/FVC. Therefore, we conclude that genetic factors account for a sizable proportion of inter-individual differences in pulmonary function, and that estimates of heritability based on SNP data are nearly identical to estimates based on pedigree data. Finally, our findings suggest a higher heritability for FEV_1_/FVC compared to either FEV_1_ or FVC.

## Introduction

Airway diseases are a major health burden, and one of the leading causes of death in the United States and worldwide (Lopez et al., 2006). Although there have been many successful efforts at identifying environmental and lifestyle risk factors (Mannino and Buist, 2007), our understanding of genetic risk factors remains limited, as it does for many complex traits, owing to multiple factors, such as an incomplete assessment of all genetic variation, and inappropriate statistical approaches (Manolio et al., 2009).

Pulmonary function as measured by spirometry serves as a diagnostic tool for diseases such as COPD and asthma. The heritability of pulmonary function, defined as the proportion of phenotypic variation that can be accounted for by genetic variation, has been estimated using twin and family studies. Estimates range from approximately 40 to 55% (Redline et al., 1989; Givelber et al., 1998; Xu et al., 1999; Wilk et al., 2000). These studies therefore suggest that genetic factors explain a substantial portion of inter-individual variation in pulmonary function. Heritability estimates using SNP-based methods, as opposed to pedigree-based methods, may allow for the accounting of variation introduced by chromosomal segregation. However, pedigree-based methods may capture more common environmental factors than captured by genetic markers.

Recent genome-wide association studies (GWAS) have identified several loci that are associated with pulmonary function and are biologically plausible candidates, such as TNS1, GSTCD, HTR4, AGER, and THSD4 (Hancock et al., 2010; Repapi et al., 2010; Weiss, 2010; Artigas et al., 2011). Although genetic variation is expected to account for approximately 50% of phenotypic variation, the loci discovered thus far account for a very small proportion of the variation in pulmonary function (Artigas et al., 2011). There is therefore a need to develop and apply methods that are capable of making use of more genetic information. Statistical methods developed in the field of animal breeding use information on thousands of genetic variants across the genome to explain phenotypic variation (Meuwissen et al., 2001). These methods have proven to be successful for production traits in livestock and plants, and have recently been shown to be useful in the context of family data for the analysis and prediction of complex human traits such as height (Makowsky et al., 2011), and less heritable traits such as lifespan (de Los Campos et al., 2012). In the case of height, heritability estimates derived using genome-wide SNP information collected in family data are essentially identical to the heritability estimate using pedigree information, and to previous estimates of height heritability based on twin and family studies (Makowsky et al., 2011). In this study, our objective is to estimate the genetic variance of pulmonary function traits by using thousands of markers distributed across the genome. Our models will be compared with those in which pedigree information is used instead.

## Methods

### Sample

Residents of Framingham, MA, USA, have been recruited since 1948 to participate in a long-term study to understand the risk factors for heart disease (Dawber et al., 1951). Spirometry testing was performed on subjects from three generations of the Framingham Heart Study. Specifically, these phenotypes were obtained from exam 19 of the Original Cohort, exams 3, 5, 6, 7, and 8 from the Offspring Cohort, and exam 1 from the Third Generation Cohort. For each Offspring cohort participant, we used in our analyses the phenotypic value from the latest examination. We included only participants who self-identified as White. A total of 6967 participants (3181 males, 3786 females) between the ages of 19 and 92 with both genotype and phenotype data were used in this analysis. We used FEV_1_ (forced expiratory volume in 1 s), FVC (forced vital capacity), and FEV_1_/FVC as the primary phenotypes of interest.

### Genotypes

Subjects were genotyped using the Affymetrix GeneChip Human Mapping 500K Array Set. For details on genotyping, see http://www.ncbi.nlm.nih.gov/projects/gap/cgi-bin/study.cgi?study_id=phs000007.v3.p2. SNPs and individuals with call rates less than 90%, as well as SNPs with a minor allele frequency (MAF) less than 0.5% were excluded. The remaining missing genotypes were imputed by sampling from a binomial distribution using the empirical MAF estimate under the assumption of Hardy-Weinberg Equilibrium. Given the low genotype missingness (approximately 1%), we do not expect that a more robust method of imputation will significantly affect the results. Genotypes from 444,938 SNPs were considered in the analysis. The first two principal components (PCs) of approximately 1000 markers that are informative for the within-Europe geographical/ancestral origin of European and European-American individuals (Drineas et al., 2010) were used as covariates in the analyses.

### Statistical models

The outcome (*y*_*i*_) (*i* = 1, …, 6967) consisted of the residual of linear regressions with FEV_1_, FVC, and FEV_1_/FVC as outcomes, and sex, age, PC1 and PC2, and cohort (to account for changes in spirometry measurement techniques) as predictors. Phenotype residuals were modeled according to an additive model of the form *y*_*i*_ = β_0_ + *u*_*i*_ + ε_*i*_ where β_0_ is an intercept, *u*_*i*_ is an additive genetic effect, representing the collective additive actions of genes potentially affecting the trait of interest, and ε_*i*_ is a component of the phenotype that cannot be explained by additive genetic effects. Stacking all the above equations from *i* = 1 to *i* = n (n = number of individuals) into vectors, we have

$$y=1\beta_{0}+u+\varepsilon$$

where **y** = (*y*_1_,…, *y*_*n*_)′, **u** = (*u*_1_,…, *u*_*n*_)′ and ε = (ε_1_,…,ε_*n*_)′ are vectors of phenotype, additive genetic effects, and model residuals, respectively.

### Pedigree model

Following the standards of the additive infinitesimal model (Fisher, 1918; Wright, 1921; Henderson, 1975), we assumed that additive genetic effects follow a multivariate normal distribution of the form **u** = **a** ~ *N* (0, A^*^σ^2^_*a*_), where σ^2^_*a*_ is an additive genetic variance and *A* is an n-dimensional matrix whose entries are pedigree-derived additive relationships (twice kinship coefficients).

### Genomic model

In this model, we replace the matrix of pedigree-derived additive relationships A, with a marker-derived estimate *G*, whose entries were: $G_{ik}=\frac{1}{p}\sum_{j\;=\;1}^{p}\frac{\left(x_{ij}-2\theta_{j}\right)\left(x_{kj}-2\theta_{j}\right)}{2\theta_{j}\left(1-\theta_{j}\right)}$, where *x*_*ij*_ is the count of allele coded as 1 for the *i*^*th*^ individual at the *j*^*th*^ SNP, *x*_*kj*_ is the count of allele coded as 1 for the *k*^*th*^ individual at the *j*^*th*^ SNP, θ_*j*_ is the estimated frequency of the allele coded as 1 at the *j*th SNP, and *p* is the number of SNPs considered (*p* = 444,938). Therefore, in this model we have: **u** = **g** ~ *N* (**0, G** * σ^2^_*g*_), where σ^2^_*g*_ is the genomic variance.

The entries of the matrix **A** give the expected patterns of genetic similarity between pairs of individuals. However, for any given pair of individuals, the expected and realized proportion of allele sharing will differ because of Mendelian sampling (Hill and Weir, 2011). The entries of the **G** matrix quantifies realized genetic similarity at markers (de los Campos et al., 2013).

In the models described above, narrow sense heritability is defined as the ratio of the genetic variance to the total variance, that is: $h_{a}^{2}=\frac{\sigma_{a}^{2}}{\sigma_{a}^{2}+\sigma_{\varepsilon }^{2}}$ for the pedigree model, and $h_{g}^{2}=\frac{\sigma_{g}^{2}}{\sigma_{g}^{2}+\sigma_{\varepsilon }^{2}}$ for the genomic model. The latter can be interpreted as the proportion of inter-individual differences in the trait of interest that can be explained by regression on common SNPs in the training sample. The parameters of the above-described model were estimated in a Bayesian framework using the BLR package (de los Campos and Pérez, 2010) in R (R Development Core Team, 2011). The variance parameters, both the residual variance and the variances of the genetic effects, were assigned an inverse chi-square distribution with scale and degree of freedom parameters equal to 2 and 5, respectively. This setting gives a relatively un-informative prior.

## Results

### Heritability of pulmonary function

The estimated coefficients for sex, age, and cohort and their statistical significance are shown in Table 1. We find a negative association between age and FEV_1_ and FVC, suggesting a significant difference between males and females, with males having higher FEV_1_ and FVC, and earlier cohorts having lower FEV_1_ and FVC. For FEV_1_/FVC, we find that females have a higher mean value than males (*p* < 5 × 10^−14^). The correlation between FEV_1_ and FVC is 0.95. However, the correlation between each of these and FEV_1_/FVC is much lower (0.46 for FEV_1_, and 0.17 for FVC).

**Table 1:** **Estimated effects and *p*-values for sex, age, and cohort in relation to three pulmonary phenotypes**.

	**FEV_1_**		**FVC**		**FEV_1_/FVC**	
	**Estimate**	***p*-value**	**Estimate**	***p*-value**	**Estimate**	***p*-value**
Sex	−0.96	<2× 10^−16^	−1.34	<2× 10^−16^	0.013	2.4 × 10^−14^
Age	−0.032	<2× 10^−16^	−0.033	<2× 10^−16^	−0.002	<2× 10^−16^
Cohort(2)	0.28	<2× 10^−16^	0.44	<2× 10^−16^	−0.006	0.021
Cohort(3)	0.44	<2× 10^−16^	0.58	<2× 10^−16^	0.007	0.095

**Figure 1:**
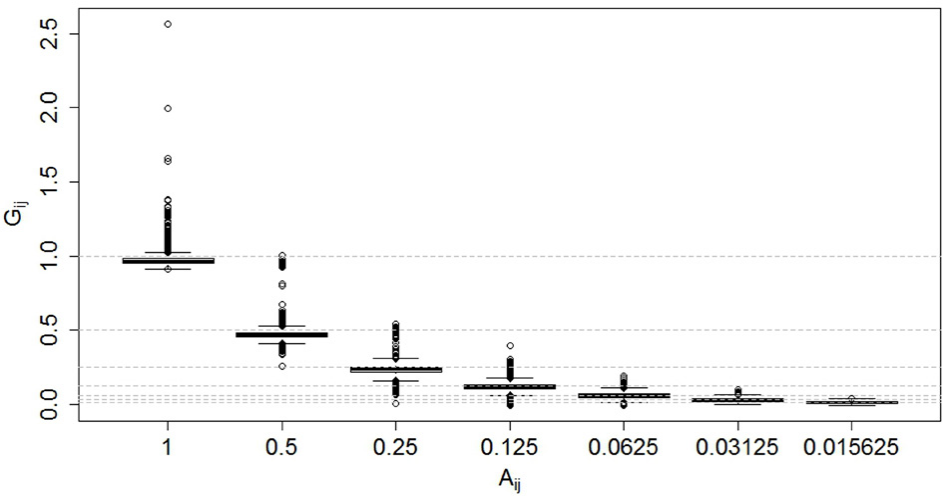
**Genomic relationship coefficients (*G_ij_*) for various levels of pedigree-based relationship coefficients (*A_ij_*).** Horizontal dashed lines indicate the different levels of expected coefficients on the y-scale.

A plot of the G-based (i.e., SNPs) relationship coefficients for different levels of A-based (i.e., pedigree) relationship coefficients is shown in Figure 1. For each level of the pedigree-based relationship coefficient, the genomic relationship coefficient varies considerably, and increasingly so at higher relationship coefficients.

Heritability estimates are shown in Table 2. Using the genomic relationship matrix, we find that approximately 50% of the variation in FEV_1_ is accounted for by the variation captured by the SNP-based relationship matrix. We obtain a slightly higher estimate when using the pedigree-based matrix. For FVC, we find that 54% of the phenotypic variation is accounted for by the SNP-based relationship matrix, while 56% is captured by the pedigree-based matrix. For FEV_1_/FVC, we find substantially higher estimates of heritability overall: 64% using the pedigree-based relationship matrix, and 66% using the SNP-based relationship matrix.

**Table 2:** **Heritability estimates and log-likelihood of models for pulmonary phenotypes based on SNP genotypes and on pedigree information (±standard error)**.

	**SNP genotypes**	**Log likelihood**	**Pedigree information**	**Log likelihood**
FEV_1_	49.75% ± 0.03	−3054.958	50.91% ± 0.03	−2873.031
FVC	53.89% ± 0.03	−4103.784	55.61% ± 0.03	−3860.512
FEV_1_/FVC	65.58% ± 0.02	10553.58	64.27% ± 0.03	10593.98

**Figure 2:**
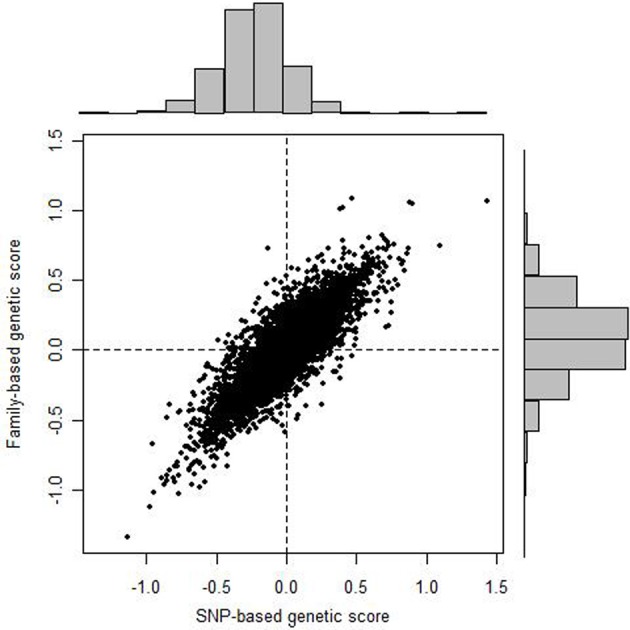
**Scatter plot of pedigree-based vs. SNP-based values of FEV_1_**.

Finally, we assessed the correspondence, on an individual level, between predicted genetic values derived from pedigree and from markers. Figure 2 shows the scatter plot of predicted values for FEV_1_. The predicted values are based on both fixed effects (sex, age, PC1, PC2, and cohort), and random effects (individual). The correlation between these estimates is approximately 0.86, 0.86, and 0.87, respectively, for FEV_1_, FVC, and FEV_1_/FVC.

## Discussion

Pulmonary function has previously been found to have a heritable basis. We examine and compare the heritability of pulmonary function using pedigree information and whole-genome SNP data. Our estimates of heritability with the SNP-based and pedigree-based methods are similar to previous estimates (Coultas et al., 1991; Ingebrigtsen et al., 2011). Interestingly the estimates for FEV_1_/FVC are considerably higher than for either FEV_1_ or FVC. It may be that by taking the ratio of FEV_1_ and FVC, much of the variation due to environmental factors is removed, compared to just FEV_1_. Indeed, it does appear that the correlation of both FEV_1_ and FVC with FEV_1_/FVC is rather low. Additionally, it may suggest a higher heritability for obstructive lung diseases such as COPD in which FEV_1_/FVC is typically reduced, as opposed to restrictive lung diseases such as fibrosis, in which FEV_1_/FVC is not typically decreased since both FEV_1_ and FVC are decreased together (Crapo, 1994; Swanney et al., 2008). A full multi-trait genetic analysis (e.g., Burgueño et al., 2012) of pulmonary phenotypes and lung diseases may provide more insight. Since we did not segregate analyses to cohorts with either restrictive or obstructive disease, another potential explanation for the higher heritability of FEV_1_/FVC is a greater genetic basis for airway dynamics and airflow for which the ratio might be a more precise measure. Such differences in heritability between FEV_1_/FVC and each of the measures on their own were not observed in previous studies (Wilk et al., 2000).

The relationship observed between the values of the A- and G-based relationship coefficients is not unexpected. We can think of realized genomic relationships as random variables whose realized values depend on the expected value (given by 1^*^kinship computed from the pedigree, *A*_*ij*_) and a deviation (d) from the expected value given by the sampling of alleles at meiosis (i.e., *G_ij_* = *A_ij_* + *d_ij_*). Therefore, the average value of *G*_*ij*_ is simply *A*_*ij*_. On the other hand, Hill and Weir (2011) showed that the variance of *G*_*ij*_ (around its mean, that is, around *A*_*ij*_) increases as *A*_*ij*_ does (simply because large chunks segregate together), and this is why we observe larger variability of *G*_*ij*_ around its mean when *A*_*ij*_ is larger.

One might expect that the SNP-based estimates of heritability would be higher than the pedigree-based estimates of heritability since the SNP information would theoretically capture information about segregation not captured by pedigree information On the other hand, pedigree-based estimates could be higher (albeit, artificially) than SNP-based estimates since pedigree information could capture more shared environmental factors than SNP information. However in this study, we find that both estimates of heritability are essentially identical, except for a slightly higher SNP-based estimate in the case of FEV_1_/FVC.

We have shown that the heritability of pulmonary phenotypes is substantial, and that the use of genome-wide SNPs in a family-based study results in essentially identical estimates of heritability as those obtained using pedigree information. Both heritability estimates could be confounded with common environmental effects that may result in inflated heritability estimates, although this is likely more of a concern in the pedigree-based estimates.

In addition to estimating overall genetic variance, the use of genome-wide SNP information also has the potential to further our understanding of the genetic basis of pulmonary function and diseases such as asthma and COPD. Given that these traits are likely highly polygenic, it will be important to continue using high-dimensional methods (both at the level of sample size and of predictors) to identify causal loci, and to better understand the genetic architecture of these traits. These causal variants are likely to be numerous and located across the genome and may be at lower frequencies than SNPs in GWAS. Improved knowledge of the genetic basis of pulmonary function could then lead to improved individualized prediction of airway disease and to targeted therapeutic options.

### Conflict of interest statement

The authors declare that the research was conducted in the absence of any commercial or financial relationships that could be construed as a potential conflict of interest.
